# A Subject for Psychological Adjudication

**Published:** 1854-04-01

**Authors:** 


### A SUBJECT FOR PSYCHOLOGICAL ADJUDICATION.

WE copy the subjoined paragraphs from the foreign correspondence of the *Times*, March 13th, and the *Observer* of March 19th. "Authentic accounts from St. Petersburg (says the *Times*) describe His Majesty to be in a state of great nervous excitement—at one time elated by the consciousness of the divine mission to extirpate infidelity and liberalism from the face of the earth, at another labouring under the greatest depression of spirits —suspecting all around him, even his most attached friends, of treachery—picturing to himself the future in the most gloomy colours, and not unfrequently fancying that his end is destined to be one of violence. He is, it appears, often oppressed with this dark cloud, and the symptoms of that malady which has more than once affected his family are evident." The following is from the *Observer:*—"All the efforts of the Russian Government tend to give to the impending struggle a character of religious ardour which is not that of 1812. Thus the Greek cross appears everywhere as the sanctifying symbol of the present war, and on every side we hear the words repeated of 'Orthodox faith,' 'Holy confidence,' 'Holy Russia,' &c. Texts from the Holy Scriptures have come to be mingled with the jargon of the fashionable saloons. The Emperor himself adopts them in conversation of the most ordinary kind, and in all his public addresses, and he appears struck with the *monomania* of preaching and haranguing to all about him in a manner that is truly ridiculous. Very recently, and in presence of his whole court, he delivered a sort of sermon, which terminated nearly with the following words:—'Russia, whose destinies God has especially entrusted to me, is menaced. But woe, woe, woe to those who menace us. We shall know how to defend the honour of the Russian name, and the inviolability of our frontier. Following in the path of my predecessors—faithful, like them, to the orthodox faith—after having invoked, like them, the aid of the Almighty God—we shall await our enemies with a firm foot, from what side soever they come, persuaded that our ancient device, "The Faith, the Czar, and the Country," will open to us, as it has ever done, the path of victory. *Nobiscum Deus, audite populi; et vincimini, quia nobiscum Deus.*' The imperial court was astounded: it never suspected that the Czar possessed this biblical erudition, and could scarcely contain its astonishment. It never suspected that his Majesty was so profoundly versed in Scripture, or in the Latin fathers. It is certain that for some time past most people are convinced that something extraordinary is the matter with the Emperor, for while his memory appears not to have failed him, his other mental faculties appear to have been seriously affected. He has become sombre and morose to an intolerable degree, either from the effect of years, or of the annoyances or embarrassments in which he sees himself placed. Perhaps all combine to produce this effect. The result is a state of exasperation which he can scarcely keep within bounds, even in presence of foreign ministers."

Would not the peace of the world have been preserved, and the valuable lives of thousands been spared, if this morbid condition of mind could have been recognised some months back, and attempts made to bring the unhappy sufferer within the range of remedial measures. It is not unreasonable to imagine that if a few leeches and blisters had been applied to the Imperial head, and his Majesty had been subjected to a course of purgation, warm bathing, and the application of the douche, the great calamity of an European war would, in all probability, have been averted, and the country rescued from the terrible infliction of an increased income tax! In a case like this the physician would have done more service than the diplomatist, and isolation and medical treatment have superseded the necessity for protocols, and have saved the Government from the expense of sendinga Queen's messenger to St. Petersburgh with the *ultimatissimum.*

---

